# Performance of Conventional Approaches Compared to Novel Blood Volume-, Angiogenesis-, and Tumor Hypoxia-Based Approaches to Diagnosing Tumor Recurrence on MRI After Radiation Therapy of Brain Metastases

**DOI:** 10.3390/diagnostics16213438

**Published:** 2026-10-22

**Authors:** Shonik Ganjoo, Samia Haffiane, Mohammed El-Aichi, Harrison Howell, Tarek Aissi, Tania Moussa, Robert Charlotte, Camilla Satragno, Corinne Balleyguier, Mabel Nuernberg, Nathalie Lassau, Gabriel Garcia, Francois Bidault, Laurent Dercle, Joya Hadchiti, Samy Ammari

**Affiliations:** 1 Vagelos College of Physicians and Surgeons, Columbia University, New York, NY 10027, USA; 2 Département d’Imagerie, Institut Gustave Roussy, Université Paris-Saclay, 94800 Villejuif, France; 3 ImmunoRadAI, Unité Mixte de Recherche (UMR) 1030 Radiothérapie Moléculaire et Innovation Thérapeutique, Institut Gustave Roussy, Inserm, Université Paris-Saclay, 94800 Villejuif, France; 4 Département de Radio-Oncologie, Institut Gustave Roussy, 94800 Villejuif, France; 5 Département d’Oncologie Medicale, Institut Gustave Roussy, Université Paris-Saclay, 94800 Villejuif, France; 6 Laboratoire BioMaps, Centre National de la Recherche Scientifique, Institut National de la Santé et de la Recherche Médicale, Commissariat à l'Énergie Atomique et aux Énergies Alternative, Université Paris-Saclay, 94800 Villejuif, France; 7 Department of Radiology, New York-Presbyterian Hospital/Columbia University Medical Center, New York, NY 10032, USA; 8 Department of Radiology, Molecular Imaging and Therapy Service, Memorial Sloan Kettering Cancer Center, New York, NY 10065, USA

**Keywords:** brain metastases, tumor recurrence, radiation necrosis, advanced magnetic resonance imaging, perfusion imaging, tumor microvasculature, oxygen metabolism, imaging biomarkers

## Abstract

**Background/Objectives:** Differentiating tumor recurrence (TR) from radiation necrosis (RN) remains a significant challenge in the post-radiation management of brain metastases due to their overlapping clinical and radiographic features. Existing imaging techniques often lack the sensitivity and specificity necessary for accurate differentiation. This study evaluates the performance of a novel suite of imaging biomarkers characterizing microvasculature, blood flow dynamics, and tumor oxygen utilization in distinguishing TR from RN, compared with established cerebral blood volume (CBV) measures. **Methods:** This retrospective study included 119 symptomatic patients with metastatic brain lesions, 7–15 months post-radiotherapy. The training cohort (*n* = 96, 182 lesions) spanned June 2013 to May 2018, while a separate test cohort (*n* = 23, 23 lesions) was collected from January 2023 to December 2024. Lesions were classified as TR or RN based on expert consensus, pathology, and long-term follow-up. Eleven imaging biomarkers were assessed in the historical cohort and nine shared biomarkers were evaluated across cohorts, including both standard and novel biomarkers. Standard biomarkers included the Apparent Diffusion Coefficient (ADC), corrected and non-corrected Cerebral Blood Volume (cCBV and ncCBV), and the Relative Amplitude of the Peak (rRHP). Novel biomarkers included Arterial Input Function Delay (Delay), Capillary Transit Time Heterogeneity (CTH and CTH Max), Oxygen Extraction Fraction (OEF), Relative Extravasation Correction (rLeakage), Relative Cerebral Metabolic Rate of Oxygen (rCMRO_2_), and Coefficient of Variance (COV). For each biomarker, a region of interest was selected from the lesion parenchyma, and a lesion-to-control parenchyma ratio was calculated. Biomarkers were evaluated for their ability to differentiate TR from RN, using the area under the receiver operating characteristic curve (AUC) for univariate analyses and random forest modeling to identify optimal biomarker combinations. Bivariate analyses modeled lesion and control parenchyma as independent variables for each biomarker and compared AUCs for models in both cohorts. **Results:** In the training cohort, novel biomarkers, including Delay (AUC = 0.994), CTH (AUC = 1.000), CTH Max (AUC = 1.000), OEF (AUC = 0.991), rCMRO_2_ (AUC = 1.000), and Coefficient of Variance (AUC = 0.993), had higher observed AUC values than cCBV (AUC = 0.930) and ncCBV (AUC = 0.786). In the test cohort, the novel biomarkers did not demonstrate a consistent advantage over cCBV or ncCBV. The random forest model identified an optimal combination of five biomarkers (cCBV, ncCBV, Delay, OEF, and COV) with an AUC of 0.964. Among these, Delay had the highest feature importance, while ncCBV contributed the least. **Conclusions:** Novel imaging biomarkers demonstrated high discrimination in the training cohort, but this performance was not reproduced in the independent test cohort. The random forest analysis identified a combination of conventional and novel biomarkers with a reported AUC of 0.964. These findings support further investigation of their potential complementary diagnostic value but remain exploratory and require validation in larger prospective multicenter cohorts.

## 1. Introduction

Radiation necrosis (RN) is a recognized late complication of radiotherapy for brain metastases and can be difficult to distinguish from tumor recurrence (TR) because their clinical and imaging findings overlap [1,2,3,4,5,6,7]. Accurate differentiation is essential because the two conditions require different management strategies. Tissue biopsy can establish the diagnosis but is invasive, motivating the development of reliable non-invasive imaging approaches [8,9].

Advanced imaging approaches include perfusion MRI, MR spectroscopy, PET, SPECT, and delayed contrast enhancement techniques [10,11,12,13,14,15,16,17]. Relative cerebral blood volume (rCBV) is widely used in clinical practice [7,12,17]. Treatment response assessment maps (TRAM) assess delayed contrast accumulation and clearance but require additional acquisition time [14,15,16]. Quantitative DSC-MRI biomarkers characterizing capillary flow heterogeneity and oxygen metabolism may provide complementary information about the tumor microenvironment [4,18,19,20,21].

Building on our previous study of conventional and TRAM-based approaches [14], the present study evaluates additional quantitative DSC-MRI biomarkers reflecting microvascular heterogeneity, perfusion dynamics, and oxygen metabolism. The historical training cohort corresponds to the population of our previous study, and a separate contemporary test cohort was added. We evaluated the individual performance of these biomarkers and their potential complementary value when combined with conventional CBV measures for differentiating TR from RN.

## 2. Materials and Methods

### 2.1. Study Design

CBV was selected as the primary comparator because it is an established and clinically relevant perfusion parameter for differentiating RN from TR and can be assessed alongside the proposed biomarkers using DSC-MRI data. The study evaluated whether the novel biomarkers could provide complementary diagnostic information relative to corrected and non-corrected CBV. To this end, the algorithm was trained on a training cohort and validated through a test cohort, as described below. We compared the predictive performance of nine distinct imaging biomarkers: corrected CBV (cCBV), non-corrected CBV (ncCBV), Delay, CTH, CTH Max, OEF, rLeakage, rCMRO_2_ and COV. cCBV is the CBV adjusted for contrast leakage due to blood–brain barrier rupture. More detailed information regarding the design and methods of this study have been previously reported elsewhere [14].

### 2.2. Study Population

A total of 205 lesions from 119 symptomatic patients were analyzed in a single-center retrospective study, conducted from June 2013 to May 2018 for the training cohort and from January 2023 to December 2024 for the test cohort. All patients were adults, and they were analyzed 7 to 15 months after having received radiotherapy for treatment of metastatic brain tumor(s). The training cohort (*n* = 96 patients with a total of 182 lesions) corresponds to the population of the previous TRAM study [14] with the addition of a test cohort (*n* = 23 patients with a total of 23 lesions). The characteristics of the study population are displayed in Table 1. All MRI scans were performed at a tertiary cancer center in France.

Inclusion criteria were as follows: (i) patients who initially responded to SRS (as indicated by a reduction in tumor size), (ii) but demonstrated signs of radiation necrosis or tumor recurrence in the following months. In all cases, a standard clinical diagnostic MRI, including T2-weighted FLAIR MRI and a pre-gadolinium T1-weighted MRI and a post-gadolinium T1-weighted MRI sequences were performed in this order: a radiotherapy planning MRI, corresponding to the initial MRI at metastasis diagnosis, followed by a contrast-enhanced MRI (for suspected radionecrosis or progression) between 7 and 15 months after SRS.

Exclusion criteria were: (i) patients in whom the MRI performed showed significant motion artifact; (ii) patients who had not received gadolinium injection; (iii) patients who had an Arterial Spin Labeling (ASL) sequence instead of the T2*-weighted echo-planar imaging sequence with first-pass gadolinium contrast.

The reference diagnosis of RN or TR was established through multidisciplinary expert assessment. The panel comprised two neuro-oncologists, three radiation therapists, and two neurosurgeons. Final diagnoses were based on MRI or choline PET-MRI findings together with clinical assessment, or on histopathological examination after surgical resection. Histopathological confirmation was available for 20 of the 96 patients in the training cohort. Additional details of the adjudication process were not systematically recorded and could not be reliably reconstructed retrospectively.

### 2.3. Imaging Parameters and Variables

MRI scanner platforms and acquisition parameters differed between the training and test cohorts, as summarized in Table 2 and Table 3. The data included at least a three-dimensional T1-weighted Fast Spoiled Gradient Recalled (FSPGR) acquisition with contrast (meglumine gadoterate [Dotarem^®^ (Guerbet, Roissy-en-France, France) , injections of 0.1 mmol/kg of gadolinium-based contrast agent]) on 3 Tesla MRI scanners (MR 750, Discovery; General Electric Healthcare, Milwaukee, WI, USA for the training cohort and IRMPremier 3T; General Electric Healthcare, Milwaukee, WI, USA in the test cohort). Imaging parameters were set as follows: repetition time: 10.2 ms; echo time: 3.4 ms; field of view: 22 cm; voxel size: 0.8 mm × 0.8 mm × 1.2 mm.

Dynamic susceptibility contrast (DSC) MRI images were acquired using a T2* weighted echo-planar imaging sequence with first-pass gadolinium contrast, comprising 50 measurements. Each measurement lasted between 1.5 and 2 s, depending on the number of slices. Continuous cross-sectional slices were taken throughout the brain for each measurement. The number of slices depended on the size of the patient’s head. The image acquisition parameters were as follows for the training cohort: repetition time: average of 2008 ms (range: 1690–2250), echo time: 62.4 ms, flip angle: 90°, slice thickness: 4 mm with 0.4 mm jump; 128 × 128 matrix. Imaging acquisition parameters for the test cohort: repetition time: average of 2400 ms, echo time: 45 ms, flip angle: 90°, slice thickness: 4 mm with a jump of 0.4 mm; 128 × 128 matrix. A series of 40 multisection acquisitions was performed at 0.2 s intervals, field of view: 140 or 150, number of excitations: 1. A bolus of paramagnetic contrast agent, gadoterate meglumine (Dotarem^®^), was given at a dose of 0.1 mmol/kg, injected at 5 mL/s, followed by a flush with 20 mL of saline solution at 3 mL/s into the cephalic venous catheter using an automatic injector (Medrad MR injection system) in both cohorts. A series of 40 multisection acquisitions was performed at 0.2-s intervals. The first 8 acquisitions were performed before the contrast agent was injected in order to establish a pre-contrast baseline.

We recorded 5 variables for the training cohort (age, type of primary tumor, whether they were treated with systemic immunotherapy, whether they underwent surgical resection of the metastatic lesion, final diagnosis of RN or TR) and 4 clinical variables for the test cohort (age, type of primary tumor, treatment with systemic immunotherapy, and final diagnosis of RN or TR). We studied 11 imaging biomarkers in the training cohort (ADC, cCBV, ncCBV, Delay, CTH, CTH Max, OEF, rLeakage, rCMRO_2_, COV, rRHP) and 9 in the test cohort (cCBV, ncCBV, Delay, CTH, CTH Max, OEF, rLeakage, rCMRO_2_ and COV).

The perfusion and diffusion plugin generated Mean Apparent Propagator (MAP) and ADC perfusion maps in relative color. Quantitative parameters were extracted as rCBV, rRHP, and PSR.

**Figure 1:**
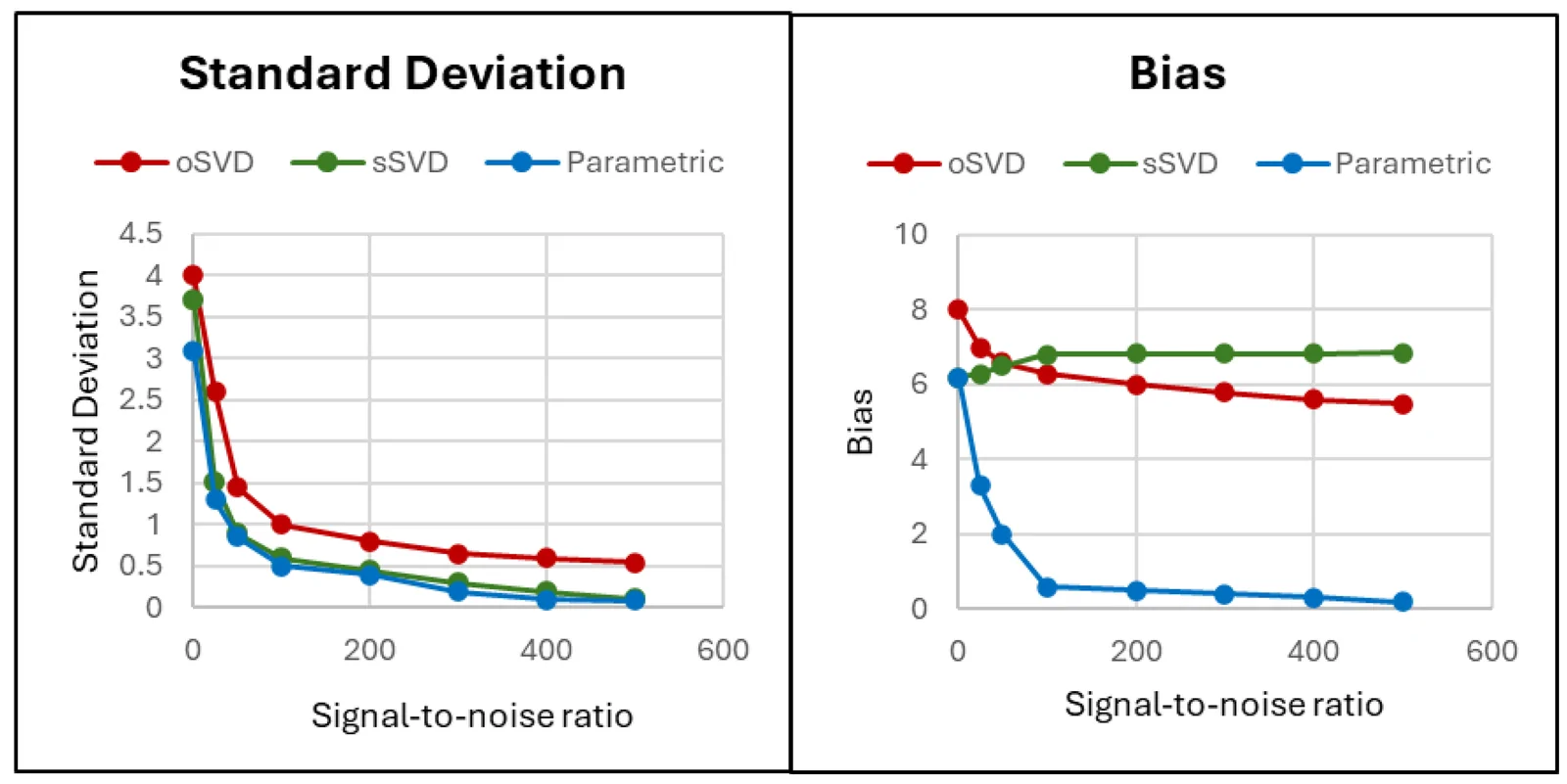
Cercare^TM^ Medical Software Imaging Maps for Novel Imaging Biomarkers: Standard Deviation (**Left**) and Bias (**Right**). oSVD = Singular Value Decomposition of Oscillatory Model. sSVD = Singular Value Decomposition of Standard Model. Data extracted from Mourisden et al. [20].

The new imaging biomarkers analyzed in our study (Delay, CTH, CTH Max, OEF, rLeakage, rCMRO_2_, COV) were extracted from the first-pass perfusion MRI sequence or Dynamic Susceptibility Contrast (DSC) MRI on Cercare^TM^ Medical (Neurosuite v16.1; Aarhus, Denmark) software and post-processed with Cercare^TM^ MRI analysis (Neurosuite v16.1; Aarhus, Denmark) software. They were presented in the form of maps based on the hypothesis that neo-angiogenesis alters the delivery of nutrients and treatments to tumor tissues, leading to a link between hemodynamic parameters and tissue oxygenation. This parametric vascular analysis model adjusts the distribution of flow and transit times through the microcirculation, providing more precise information on blood flow behavior. The maps generated showed less noise and fewer artifacts by taking into account flow heterogeneity and potential leakage, and by using Bayesian statistics to better reflect true global perfusion (Figure 1).

As demonstrated above, the parametric technique is able to produce independent estimates of MTT and CTH, with a reduction in model bias as the signal-to-noise ratio (SNR) increases. This is in contrast to the singular value decomposition (SVD) model, which does not account for the physiological distribution of tissue transit times. Such an approach fails to accurately model the dispersion and delay of the contrast agent, particularly in regions where cerebral flow is complex or delayed [20]. In the SVD model, calculations vary according to capillary transit time heterogeneity, with a model bias that is not improved with increasing SNR (Figure 1).

For each biomarker, we computed three values based on region of interest (ROI): path (corresponding to ROI in the lesion), control (corresponding to ROI in the contralateral parenchyma), and ratio (ratio of path/control). Notably, the ratio normalizes for inter-subject variability, demonstrates superior predictive capability compared to path alone, and has comparable predictive performance to bivariate models when used alone in a univariate model [22,23]. Therefore, we used the AUC values from univariate analyses evaluating the ratio of each novel sequence to establish predictive performance.

### 2.4. Image Analysis

Tumor segmentation: Segmentation of the volume of interest, including the contrast-enhanced and necrotic regions, was performed semi-automatically using Cercare^TM^ Medical software. Regions of interest were defined by a trained radiologist (with 5+ years of experience), and threshold-based grey level contouring and manual correction were used for the segmentation such that the volume of interest was carefully drawn along the tumor enhancement.

### 2.5. Random Forest Model Construction

A supervised machine learning model using the random forest algorithm was constructed to evaluate the diagnostic performance of the established rCBV biomarker in combination with the novel biomarkers. The initial pool of biomarkers included cCBV, ncCBV, CTH, CTH Max, rCMRO_2_, rLeakage, OEF, COV, and Delay. We implemented a random forest classifier using the scikit-learn library in Python (v0.20.3) [24]. A grid search was used to optimize the following hyperparameters: Number of trees (n_estimators): [10, 20, 50, 100]; Maximum tree depth (max_depth): [1, 2, 3, 4, 5, None]; Number of features considered at each split (max_features): [1 to N (number of biomarkers), None]; Bootstrap sampling (bootstrap): [True, False]; Minimum samples to split a node (min_samples_split): [2, 5, 10]; Minimum samples required at each leaf node (min_samples_leaf): [1, 2, 4, 6]. The optimal parameter combination was identified as: n_estimators = 20, max_depth = 3, max_features = 1, bootstrap = True, min_samples_split = 5, min_samples_leaf = 1. COV and rCMRO_2_ were specified a priori and retained as physiologically meaningful composite biomarkers. COV is derived as CTH/MTT, and rCMRO_2_ is derived from OEF × CBF. Their mathematical relationships with related physiological parameters may introduce redundancy when these measures are evaluated together. MTT and CBF were not included as separate model predictors. The selected five-feature model did not include CTH or rCMRO_2_.

Random forest models used lesion-to-control biomarker ratios as predictors and the reference diagnosis of TR or RN as the outcome. Model discrimination was assessed using AUC in the training and independent test cohorts. Feature importance was computed using the mean decrease in Gini impurity, as implemented in the feature_importances_ attribute of the random forest model. The random forest model was trained exclusively on the training cohort and subsequently evaluated on the independent test cohort without further model fitting or parameter optimization.

### 2.6. Statistical Analysis

Statistical analysis was performed using R software (v3.6.2). AUC was used to assess the diagnostic performance of univariate models incorporating each biomarker. Bivariate analyses included bivariate logistic regression models, SVM (linear) bivariate models, and SVM (RBF) bivariate models. Each of the bivariate analyses used AUC values to assess the diagnostic performance of the path and control variables. Bivariate analyses were included as exploratory analyses to determine whether separate modeling of lesion and contralateral control measurements provided additional diagnostic information beyond the lesion-to-control ratio alone. For the SVM bivariate models specifically, two AUC values were computed: decision function (margin) and predicted probability (proba). Margin refers to the raw output of the SVM decision function, indicating how far a sample lies from the decision boundary and is used directly for classification. Proba refers to a calibrated probability estimate obtained by applying Platt scaling, fitting a sigmoid function to the SVM margins.

## 3. Results

### 3.1. Univariate Analyses

#### 3.1.1. Training Data

**Figure 2:**
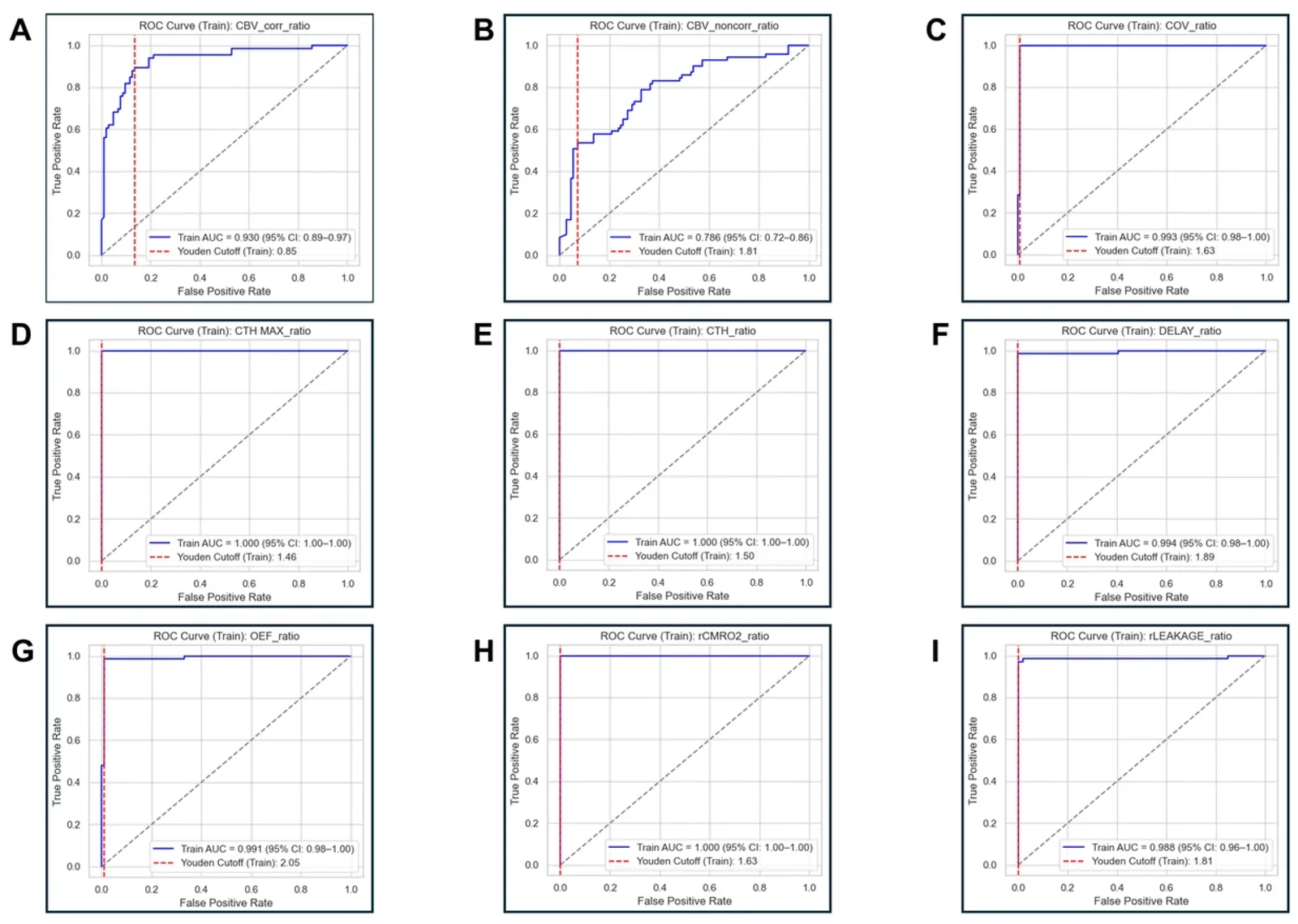
Training ROC Curves from Univariate Models Incorporating the Ratio of Nine Investigated Biomarkers. (**A**) cCBV_ratio (**B**) ncCBV_ratio (**C**) COV_ratio (**D**) CTH Max_ratio (**E**) CTH_ratio (**F**) Delay_ratio (**G**) OEF_ratio (**H**) rCMRO_2_ ratio (**I**) rLeakage_ratio. Grey dashed lines indicate chance-level discrimination (AUC =0.5). ROC, receiver operating characteristic; AUC, area under the ROC curve.

Across the univariate models, Delay_ratio (AUC = 0.994; 95% CI: 0.983–1.000), CTH_ratio (AUC = 1.000; 95% CI: 1.000–1.000), CTH Max_ratio (AUC = 1.000; 95% CI: 1.000–1.000), OEF_ratio (AUC = 0.99; 95% CI: 0.978–1.000), rCMRO_2__ratio (AUC = 1.000; 95% CI: 1.000–1.000), and COV_ratio (AUC = 0.993; 95% CI: 0.981–1.000) all had higher observed AUC values than cCBV_ratio (AUC = 0.930; 95% CI: 0.890–0.971) and ncCBV_ratio (AUC = 0.786; 95% CI: 0.717–0.856). Additionally, rLeakage (AUC = 0.988; 95% CI: 0.964–1.000) had a higher observed AUC than ncCBV_ratio and cCBV_ratio (Figure 2 Table S1). These training-cohort estimates are exploratory and should not be interpreted as definitive evidence of superiority over CBV.

#### 3.1.2. Test Data

**Figure 3:**
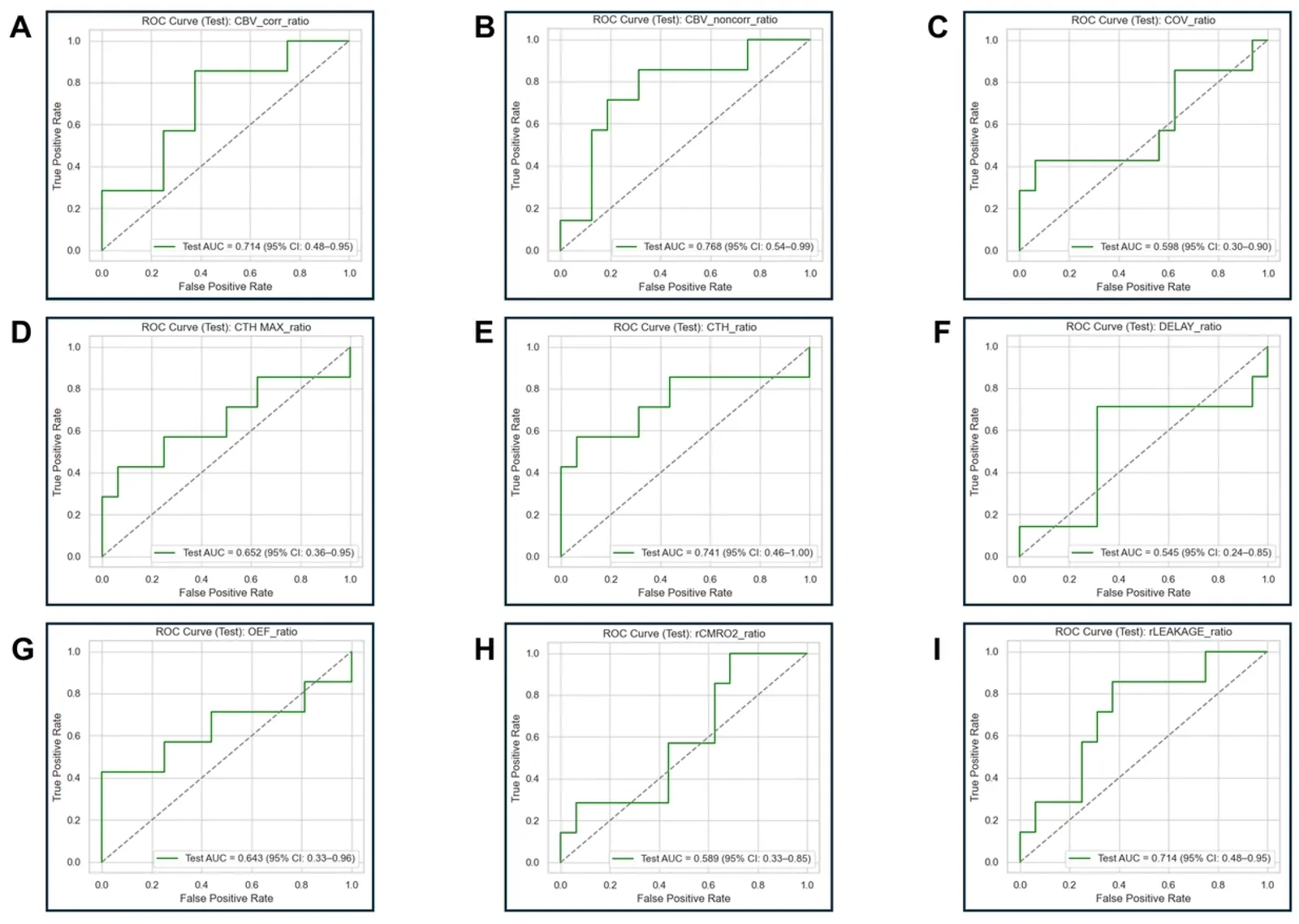
Test ROC Curves from Univariate Models Incorporating the Ratio of Nine Investigated Biomarkers. (**A**) cCBV_ratio (**B**) ncCBV_ratio (**C**) COV_ratio (**D**) CTH Max_ratio (**E**) CTH_ratio (**F**) Delay_ratio (**G**) OEF_ratio (**H**) rCMRO_2_ ratio (**I**) rLeakage_ratio. Grey dashed lines indicate chance-level discrimination (AUC =0.5). ROC, receiver operating characteristic; AUC, area under the ROC curve.

**Figure 4:**
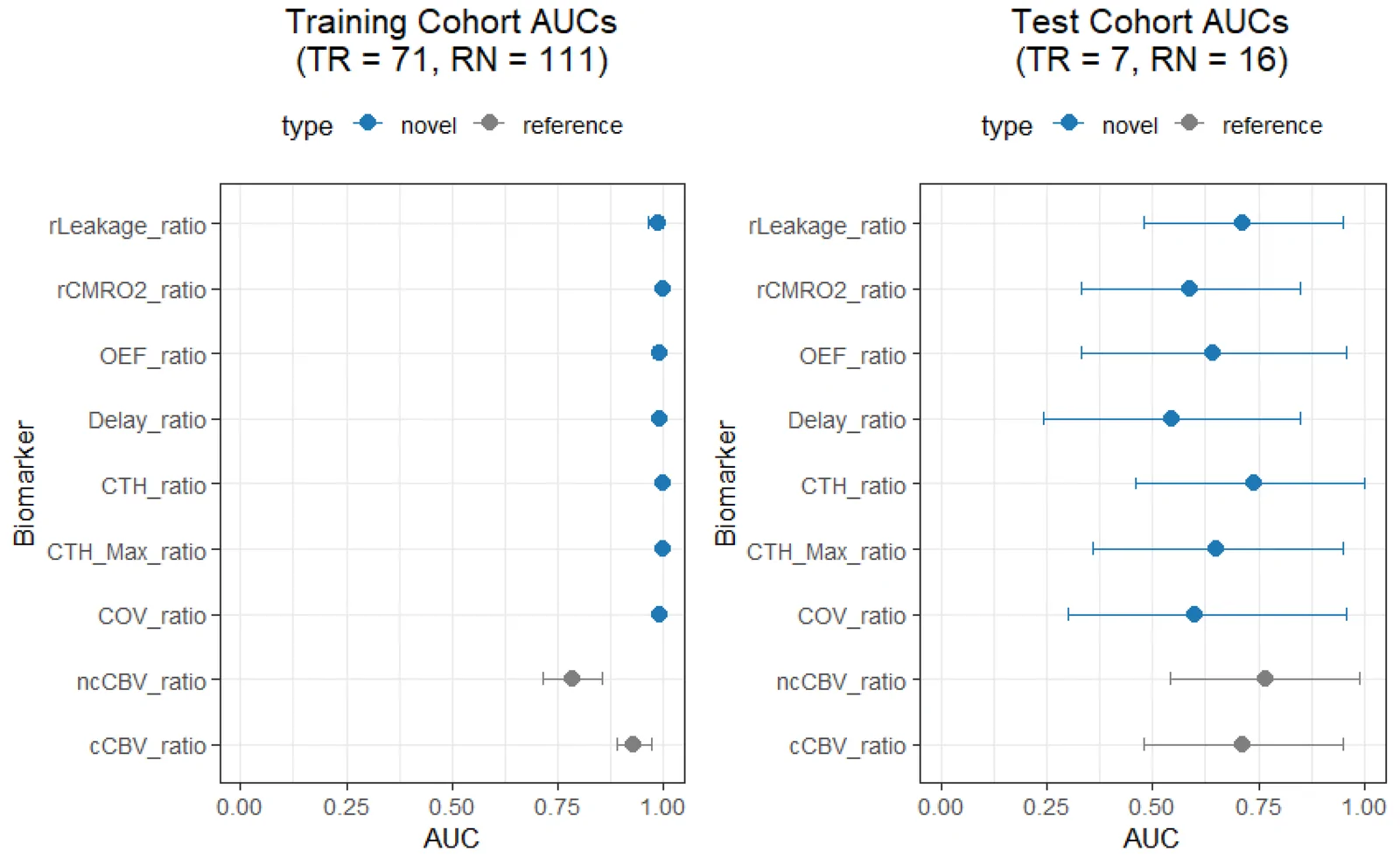
Discrimination of Individual Biomarker Ratios in the Treaining and Test Cohorts.

Across the univariate models, Delay_ratio (AUC = 0.545; 95% CI: 0.24–0.85), CTH_ratio (AUC = 0.741; 95% CI: 0.46–1.00), CTH Max_ratio (AUC = 0.652; 95% CI: 0.36–0.95), OEF_ratio (AUC = 0.643; 95% CI: 0.33–0.96), rCMRO_2__ratio (AUC = 0.589; 95% CI: 0.33–0.85), COV_ratio (AUC = 0.598; 95% CI: 0.3–0.9), and rLeakage_ratio (AUC = 0.714; 95% CI: 0.48–0.95) did not demonstrate a consistent advantage over cCBV_ratio (AUC = 0.714; 95% CI: 0.48–0.95) and ncCBV_ratio (AUC = 0.768; 95% CI: 0.54–0.99) (Figure 3 and Figure 4, Table S1). The small test cohort and wide confidence intervals limit the precision of these estimates and do not establish equivalence between biomarkers.

#### 3.1.3. Logistic Regression

Univariate logistic regression models incorporating the ratios for each biomarker revealed that cCBV_ratio (OR = 8.35, *p* < 0.001), ncCBV_ratio (OR = 2.63, *p* < 0.001), Delay_ratio (OR = 72.37, *p* < 0.001), OEF_ratio (OR = 12.08, *p* < 0.001), rLeakage_ratio (OR = 285.69, *p* = 0.01), and COV_ratio (OR = 55.56, *p* < 0.001) were all significant predictors of TR. The ORs for CTH_ratio (OR = 9.78 × 1026, *p* = 1.00), CTH Max_ratio (5.10 × 1026, *p* = 1.00), and rCMRO_2__ratio (9.89 × 1015, *p* = 1.00) were found to be exceptionally large due to potential overfitting or perfect separation in the model (Table 4). For CTH_ratio, CTH Max_ratio, and rCMRO_2__ratio, complete separation resulted in unstable logistic regression coefficient estimates and non-informative confidence intervals. ROC-based discrimination estimates are reported alongside these unstable regression estimates; however, the near-perfect training-cohort AUC values also require cautious interpretation and independent validation.

### 3.2. Bivariate Analyses

#### 3.2.1. Bivariate Logistic Regression Models

In comparison to the corresponding univariate models using the ratio for each biomarker, the cCBV_path + cCBV_control (AUC = 0.714; 95% CI: 0.474–0.954), ncCBV_path + ncCBV_control (AUC = 0.821; 95% CI: 0.599–1.000), CTH_path + CTH_control (AUC = 0.786; 95% CI: 0.500–1.000), CTH Max_path + CTH Max_control (AUC = 0.714; 95% CI: 0.420–1.000), and OEF_path + OEF_control (AUC = 0.705; 95% CI: 0.395–1.000) models demonstrated comparable predictive performance. However, the Delay_path + Delay_control (AUC = 0.580; 95% CI: 0.258–0.903), rLeakage_path + rLeakage_control (AUC = 0.679; 95% CI: 0.427–0.930), rCMRO_2__path + rCMRO_2__control (AUC = 0.527; 95% CI: 0.286–0.767), and COV_path + COV_control (AUC = 0.607; 95% CI: 0.309–0.906) models demonstrated significantly lower predictive ability than the univariate models (Table 5).

#### 3.2.2. SVM (Linear) Bivariate Models

Based on margin-based probability evaluations and Platt-scaled probability evaluations, the AUC values and 95% CIs from SVM linear bivariate models incorporating the path and control for each biomarker were nearly identical to those found in the bivariate logistic regression model (Table 5). The correlation between these models was r = 0.996.

#### 3.2.3. SVM (RBF) Bivariate Models

Based on margin-based probability evaluations, the cCBV_path + cCBV_control (AUC = 0.714; 95% CI: 0.474–0.954), ncCBV_path + ncCBV_control (AUC = 0.652; 95% CI: 0.386–0.918), and OEF_path + OEF_control (AUC = 0.688; 95% CI: 0.371–1.000) models demonstrated comparable predictive performance to the corresponding univariate models incorporating each biomarker. On the other hand, the Delay_path + Delay_control (AUC = 0.598; 95% CI: 0.270–0.926), CTH_path + CTH_control (AUC = 0.598; 95% CI: 0.326–0.871), CTH Max_path + CTH Max_control (AUC = 0.321; 95% CI: 0.094–0.549), rLeakage_path + rLeakage_control (AUC = 0.607; 95% CI: 0.329–0.885), rCMRO_2__path + rCMRO_2__control (AUC = 0.536; 95% CI: 0.294–0.778), and COV_path + COV_control (AUC = 0.598; 95% CI: 0.297–0.900) models demonstrated significantly lower predictive probability than the univariate models (Table 5).

Based on Platt-scaled probability evaluations, the AUC values and 95% CIs were identical to those of the margin-based probability evaluations, and there were therefore no significant differences in predictive performance between margin-based probability evaluations and Platt-scaled probability evaluations for any of the nine biomarkers in SVM (RBF) bivariate models.

### 3.3. Comparison Between Univariate and Bivariate Models

**Figure 5:**
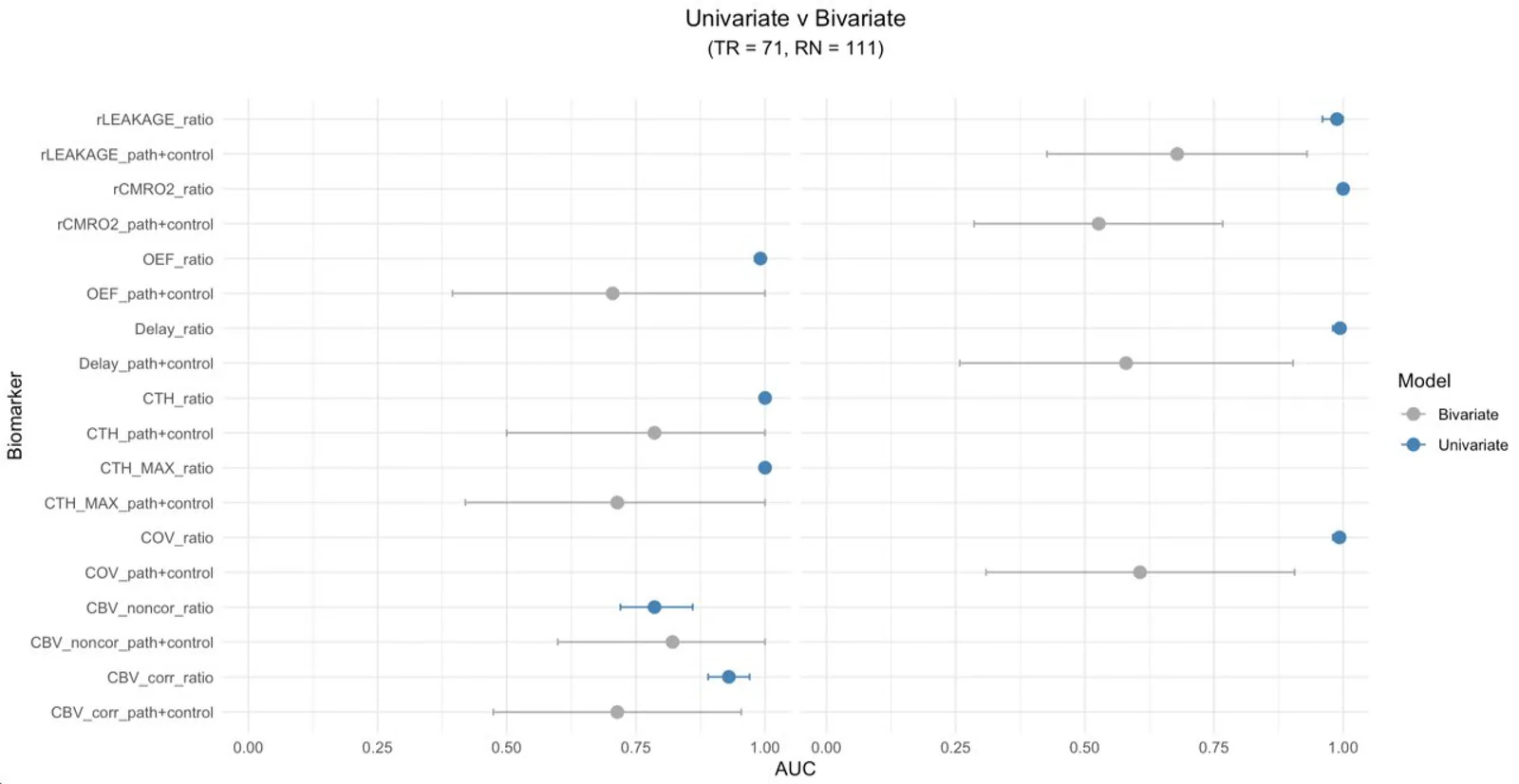
Comparison of Predictive Model Efficiency Between Univariate Model Incorporating the Ratio of Each Biomarker Versus Bivariate Model Incorporating the Path and Control for Each Biomarker. (**Left**): Non-significant Differences Between Univariate and Bivariate Models; (**Right**): Significant Differences Between Univariate and Bivariate Models.

The univariate model incorporating the ratio of each biomarker was overall more efficient in predictively modeling TR vs. RN compared to the bivariate approach for all biomarkers (Figure 5).

### 3.4. Random Forest Model

**Figure 6:**
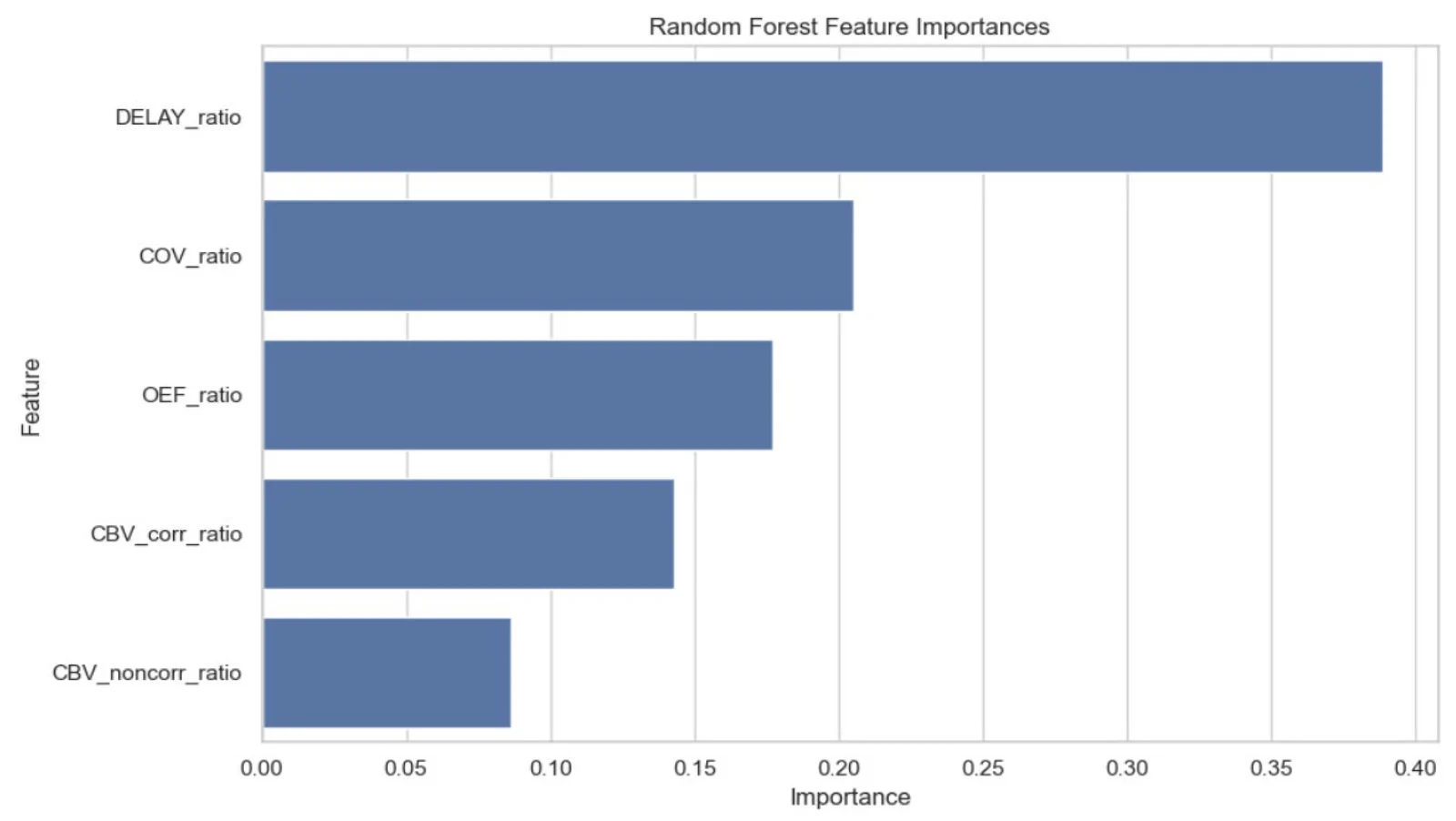
Random Forest Feature Importances Based on Optimal Model Construction Using cCBV_ratio, ncCBV_ratio, Delay_ratio, OEF_ratio, and COV_ratio.

The optimal model after performing a grid search using a subset of five biomarkers was: cCBV_ratio, ncCBV_ratio, Delay_ratio, OEF_ratio, and COV_ratio [AUC = 0.964 (95% CI: 0.898–1.000)]. Delay_ratio demonstrated the highest relative feature importance in this model, while cCBV_ratio and ncCBV_ratio demonstrated the lowest relative feature importances (Figure 6).

## 4. Discussion

This study evaluated quantitative DSC-MRI biomarkers of microvascular heterogeneity and oxygen metabolism alongside conventional CBV measures in patients with irradiated brain metastases. Several biomarkers showed high discrimination in the historical training cohort, particularly CTH_ratio, CTH Max_ratio, and rCMRO_2__ratio. Their substantially lower performance in the separate contemporary test cohort demonstrates that these training-cohort findings did not translate consistently across the two cohorts. The study therefore identifies promising biomarker signals while providing an initial assessment of their reproducibility under different acquisition and cohort conditions.

The clinical motivation is the need to distinguish active tumor recurrence from radiation necrosis after radiotherapy for brain metastases, because these diagnoses require different management strategies [4,5]. DSC-MRI-derived measures of microvascular function and oxygen metabolism offer a physiological basis for investigating this distinction. The present findings inform the selection and evaluation of candidate biomarkers for subsequent validation.

Elevated CTH can result from abnormal capillary bed topology, arteriovenous shunts, microthrombosis, pericyte loss, or increased interstitial pressure due to edema [25]. Our findings are consistent with theoretical perspectives presented in reviews discussing the potential impact of altered capillary morphology in tumors [26,27], where CTH is elevated in the disorganized microvasculature and in areas of extensive angiogenesis typically present in the tumor environment [28]. In addition, consistent with the Warburg effect, elevated CTH differentially impairs the extraction of oxygen and glucose, thereby modulating the degree of aerobic glycolysis [26]. As expected, increased CTH in combination with high blood flow leads to reduced oxygen extraction and local hypoxia [29].

This postulation is substantiated by previous findings demonstrating increased expression of hypoxia-related markers, such as HIF-1α and VEGF, in tissue regions characterized by altered capillary transit times [29,30]. It is well established that tumor progression and resistance to both radiotherapy and chemotherapy are linked to a hypoxic tumor microenvironment, commonly referred to as the “hypoxic niche” near capillaries, where they act as the source of growing malignant tissue [31]. This link may be due to tumor-initiating stem cells’ preference for low-oxygen (hypoxic) conditions [32,33].

Moreover, in a typical capillary network, CTH generally rises in proportion to the MTT, resulting in COV maps that display minimal contrast in healthy tissue [28,34]. However, in tumor tissue, these 2 parameters may be uncoupled, leading to high COV in TR compared to RN, which has been shown previously [28].

Higher rCMRO_2_ values were observed in the TR group in the training cohort. As a measure related to oxygen metabolism, rCMRO_2_ provides a physiological rationale for investigating differences between recurrent tumor and radiation necrosis. Its diagnostic performance, however, was substantially lower in the contemporary test cohort. Previous work has also suggested that reliance on rCMRO_2_ alone may overlook potential sites of recurrence because of altered tumor metabolism, including the Warburg effect [35,36].

In the training cohort, ncCBV_ratio had the lowest observed AUC among the evaluated biomarkers (0.786). Although rCBV is widely used to distinguish TR from RN, previous studies have reported variability in its diagnostic performance and overlapping values between the two conditions [17,37]. This overlap may be attributed to several factors, including tumor heterogeneity, the co-existence of radionecrotic lesions with tumor recurrence, and the presence of radionecrotic tissue within samples taken from areas of metastatic recurrence. Additionally, magnetic susceptibility artifacts—often caused by petechial hemorrhages following irradiation—can artificially lower rCBV values, even in cases of true tumor recurrence [7,37].

The ratio-based and separate lesion–control approaches represent different ways of incorporating an internal reference measurement. The ratio directly expresses lesion contrast relative to contralateral tissue, whereas bivariate models estimate the contributions of the two measurements separately. Performance varied by biomarker and model specification. The present exploratory comparisons do not establish a universally preferable modeling approach.

The exploratory random forest analysis identified a selected combination of five biomarkers (cCBV_ratio, ncCBV_ratio, Delay_ratio, OEF_ratio, and COV_ratio) for differentiating TR from RN. Variable importance estimates from random forest models should be interpreted cautiously, as Gini impurity–based measures are biased toward continuous variables and those with more potential split points. Consequently, importance rankings may not reflect true independent or causal effects. In this study, variable importance is therefore used for hypothesis generation rather than to establish definitive biomarker relevance.

Delay_ratio had the highest feature importance in the selected random forest model, but its standalone performance was substantially lower in the independent test cohort. This discrepancy may reflect optimistic training estimates, differences in cohort characteristics or acquisition conditions, or a combination of these factors. The small test cohort further limits precision, and the present data cannot determine the relative contribution of these explanations.

Other clinically relevant approaches include MR spectroscopy, metabolic imaging with PET or SPECT [10,11,38], and delayed contrast enhancement techniques such as TRAM [14,15,16]. CBV was selected as the primary comparator because it is an established perfusion measure obtained from DSC-MRI. The present study did not directly compare the proposed biomarkers with these other modalities or evaluate their incremental value within a multimodal diagnostic strategy. Their potential role should therefore be considered complementary and investigational.

Several limitations should be considered. This single-center retrospective study included heterogeneous primary tumor types and a small contemporary test cohort of 23 patients. The resulting uncertainty limits conclusions about comparative performance and generalizability. Although confidence intervals are reported for AUC estimates, uncertainty estimates are not available for every performance metric.

The training cohort included 182 lesions from 96 patients, and analyses did not account for within-patient clustering, which may affect statistical precision. Near-perfect discrimination also produced complete or quasi-complete separation in some logistic regression analyses. The observed training–test performance gap may reflect optimistic training estimates, cohort differences, acquisition differences, or a combination of these factors; their relative contributions cannot be determined from the present analyses.

Diagnoses were established using a composite reference standard, with histopathological confirmation available for 20 training-cohort patients. Incomplete adjudication documentation and the absence of pathology-restricted analyses limit assessment of misclassification and incorporation bias. Detailed treatment information was also incomplete, preventing a comprehensive assessment of temporal treatment-related confounding.

Scanner platforms and acquisition parameters differed between cohorts, and cross-scanner harmonization was not performed. Proprietary processing requirements further limit transferability. Model evaluation focused primarily on discrimination; calibration, stability, and clinical utility require further assessment. Related and composite biomarkers may also complicate interpretation of individual feature contributions, which were not assessed through additional feature-exclusion analyses.

These limitations define the study as an exploratory assessment rather than evidence for routine clinical implementation. Further evaluation should incorporate patient-level clustering, standardized acquisition and processing, more complete reference-standard and treatment documentation, and independent multicenter validation.

## 5. Conclusions

Quantitative DSC-MRI biomarkers of microvascular heterogeneity and oxygen metabolism showed high discrimination between tumor recurrence and radiation necrosis in the historical training cohort, but their individual performance was substantially lower in the contemporary test cohort. An exploratory random forest analysis identified a combination of conventional and novel biomarkers. Together, these findings support continued investigation of physiologically informed DSC-MRI measures and highlight the importance of independent validation across acquisition conditions and patient populations. Larger prospective multicenter studies are needed to determine their incremental diagnostic value.

## Figures and Tables

**Table 1:** Characteristics of the Study Population.

Characteristic	Parameters and Values in Training Cohort (*n* = 182)	Parameters and Values in Test Cohort (*n* = 23)
Age (years)	Mean: 60	Mean: 62
Sex	Female: 81 (45%); Male: 101 (55%)	Female: 12 (52%); Male: 11 (48%)
Primary Tumor	Bronchial Tumor: 81 (45%); Melanoma: 48 (26%); Breast Carcinoma: 25 (14%); Papillary Thyroid Carcinoma: 9 (5%); Ovarian Tumor: 6 (3%); Sarcoma: 8 (4%); Other: 5 (2%)	Bronchial Tumor: 8 (35%); Melanoma: 2 (9%); Breast Carcinoma: 7 (30%); Papillary Thyroid Carcinoma: 2 (9%); Ovarian Tumor: 1 (4%); Clear Cell Kidney Tumor: 1 (4%); Sarcoma: 0 (0%); Other: 2 (9%)
Systemic treatment: Immunotherapy?	Yes: 72 (40%); No: 110 (60%)	Yes: 6 (26%); No: 17 (74%)
Final Diagnosis	Radionecrosis: 111 (61%); Tumor Recurrence: 71 (39%)	Radionecrosis: 16 (70%); Tumor Recurrence: 7 (30%)
Surgical Intervention?	Yes: 20 (11%); No: 162 (89%)	N/A

**Table 2:** MRI Parameters Using Discovery MR750w 3T for the Training Cohort.

Sequence Name	Sequence Type	Repetition Time (ms)	Echo Time (ms)	Cut Thickness (mm)
T1 Pre-Contrast	3D Rapid Gradient Echo	5.9	2.1	1
T2-Flair	Turbo Spin Echo	7002	118	1
DWI	EPI, Two b-values (0 and 1000 mm^2^/s)	5375	62.6	3
Dynamic Susceptibility MRI (DSC)	First Pass Gadolinium Contrast-Enhanced T2*-Weighted EPI	2008 (Range: 1690–2250)	62.4	4
T1 Post-Contrast 3 min After Injection	3D Rapid Gradient Echo	6.1	2.1	1
T1 Post-Contrast 70 min After Injection	3D Rapid Gradient Echo	6.1	2.1	1

**Table 3:** MRI Parameters Using MRPremier 3T for the Test Cohort.

Sequence Name	Sequence Type	Repetition Time (ms)	Echo Time (ms)	Cut Thickness (mm)
T1 Pre-Contrast	3D Rapid Gradient Echo	Minimum	In Phase	0.6
T2-Flair	Turbo Spin Echo	7800	120	0.6
DWI	EPI, Two b-values (0 and 1000 mm^2^/s)	5972	Minimum	3
Dynamic Susceptibility MRI (DSC)	First Pass Gadolinium Contrast-Enhanced T2*-Weighted EPI	2400	45	4
T1 Post-Contrast 3 Minutes After Injection	3D Rapid Gradient Echo	500	Minimum	0.6
T1 Post-Contrast 70 Minutes After Injection	3D Rapid Gradient Echo	Minimum	In Phase	0.6

**Table 4:** Univariate Logistic Regression Model Incorporating Ratio of Each Biomarker.

Biomarker	Odds Ratio (OR)	95% CI for OR	*p*-Value
cCBV_ratio	8.35	(4.38, 15.91)	*p* < 0.001
ncCBV_ratio	2.63	(1.81, 3.83)	*p* < 0.001
Delay_ratio	72.37	(8.87, 590.43)	*p* < 0.001
CTH_ratio	9.78 × 10^26^	(0, inf)	*p* = 1.00
CTH Max_ratio	5.10 × 10^26^	(0, inf)	*p* = 1.00
OEF_ratio	12.08	(5.28, 27.64)	*p* < 0.001
rLeakage_ratio	285.69	(3.49, 23,375.87)	*p* = 0.01
rCMRO_2__ratio	9.89 × 10^15^	(0, inf)	*p* = 1.00
COV_ratio	55.56	(13.62, 226.70)	*p* < 0.001

**Table 5:** Bivariate Models Incorporating Path ROI and Control ROI for Biomarkers as Predictors: AUC values in Training Cohorts.

Biomarker	Bivariate Logistic Regression AUC (95% CI)	Margin-Based SVM (Linear) Bivariate Model AUC (95% CI)	Platt-Scaled SVM (Linear) Bivariate Model AUC (95% CI)	Margin-Based SVM (RBF) Bivariate Model AUC (95% CI)	Platt-Scaled SVM (RBF) Bivariate Model AUC (95% CI)
CTH_path + CTH_control	0.786 (0.500–1.000)	0.786 (0.500–1.000)	0.786 (0.500–1.000)	0.598 (0.326–0.871)	0.598 (0.326–0.871)
CTH Max_path + CTH Max_control	0.714 (0.420–1.000)	0.714 (0.420–1.000)	0.714 (0.420–1.000)	0.321 (0.094–0.549)	0.321 (0.094–0.549)
rCMRO_2__path + rCMRO_2__control	0.527 (0.286–0.767)	0.536 (0.294–0.778)	0.536 (0.294–0.778)	0.536 (0.294–0.778)	0.536 (0.294–0.778)
Delay_path + Delay_control	0.580 (0.258–0.903)	0.562 (0.246–0.879)	0.562 (0.246–0.879)	0.598 (0.270–0.926)	0.598 (0.270–0.926)
COV_path + COV_control	0.607 (0.309–0.906)	0.598 (0.296–0.900)	0.598 (0.296–0.900)	0.598 (0.297–0.900)	0.598 (0.297–0.900)
OEF_path + OEF_control	0.705 (0.395–1.000)	0.696 (0.378–1.000)	0.696 (0.378–1.000)	0.688 (0.371–1.000)	0.688 (0.371–1.000)
rLeakage_path + rLeakage_control	0.679 (0.427–0.930)	0.679 (0.427–0.930)	0.679 (0.427–0.930)	0.607 (0.329–0.885)	0.607 (0.329–0.885)
cCBV_path + cCBV_control	0.714 (0.474–0.954)	0.714 (0.476–0.953)	0.705 (0.467–0.944)	0.714 (0.474–0.954)	0.714 (0.474–0.954)
ncCBV_path + ncCBV_control	0.821 (0.599–1.000)	0.812 (0.589–1.000)	0.812 (0.589–1.000)	0.652 (0.386–0.918)	0.652 (0.386–0.918)

## Data Availability

The original contributions presented in this study are included in the article/Supplementary Materials . Further inquiries can be directed to the corresponding author.

## Supplementary Materials

The following supporting information can be downloaded at: https://www.mdpi.com/article/10.3390/diagnostics16213438/s1, Table S1: Numerical AUC Estimates and 95% Confidence Intervals for Individual Biomarker Ratios in the Training and Test Cohorts.

## Abbreviations

The following abbreviations are used in this manuscript:

TR	Tumor Recurrence
RN	Radiation Necrosis
CBV	Cerebral Blood Volume
ADC	Apparent Diffusion Coefficient
cCBV	Corrected Cerebral Blood Volume
ncCBV	Non-corrected Cerebral Blood Volume
rRHP	Relative Amplitude of the Peak
AIF	Arterial Input Function
CTH	Capillary Transit Time Heterogeneity
OEF	Oxygen Extraction Fraction
rLeakage	Relative Extravasation Correction
rCMRO_2_	Relative Cerebral Metabolic Rate of Oxygen
COV	Coefficient of Variance
AUC	Area Under Receiver Operating Characteristic Curve
MRI	Magnetic Resonance Imaging
PET	Positron Emission Tomography
SPECT	Single-Photon Emission Computed Tomography
TRAM	Late Enhancement Sequence
PSR	Percentage Signal Recovery
SRS	Stereotactic Radiosurgery
ASL	Arterial Spin Labeling
DSC	Dynamic Susceptibility Contrast
MAP	Mean Apparent Propagator
SNR	Signal-to-Noise Ratio
SVD	Singular Value Decomposition
ROI	Region of Interest
SVM	Support Vector Machine
RBF	Radial Basis Function
MRS	Magnetic Resonance Spectroscopy
DSC-PWI	Dynamic Susceptibility Contrast Perfusion-Weighted Imaging
HIF-1α	Hypoxia-Inducible Factor-1α
VEGF	Vascular Endothelial Growth Factor
