# Supplementary material for: Performance of Conventional Approaches Compared to Novel Blood Volume-, Angiogenesis-, and Tumor Hypoxia-Based Approaches to Diagnosing Tumor Recurrence on MRI After Radiation Therapy of Brain Metastases
Source: Diagnostics (Basel). 2026 Oct 22;16(21):3438. doi: 10.3390/diagnostics16213438 (PMC13761481; doi:10.3390/diagnostics16213438)
Supplement: Supplementary file 1 [file diagnostics-16-03438-s001.zip › diagnostics-4527922-supplementary.pdf]

Table S1: Numerical AUC Estimates and 95% Confidence Intervals for Individual Biomarker Ratios in the Training and Test Cohorts.

Biomarker	Training AUC (95% CI)	Test AUC (95% CI)
CTH_ratio	1.000 (1.000 – 1.000)	0.741 (0.46 – 1.00)
CTH Max_ratio	1.000 (1.000 – 1.000)	0.652 (0.36 – 0.95)
$rCMRO_2$_ratio	1.000 (1.000 – 1.000)	0.589 (0.33 – 0.85)
Delay_ratio	0.994 (0.983 – 1.000)	0.545 (0.24 – 0.85)
COV_ratio	0.993 (0.981 – 1.000)	0.598 (0.3 – 0.9)
OEF_ratio	0.991 (0.978 – 1.000)	0.643 (0.33 – 0.96)
rLeakage_ratio	0.988 (0.964 – 1.000)	0.714 (0.48 – 0.95)
cCBV_ratio	0.930 (0.890 – 0.971)	0.714 (0.48 – 0.95)
ncCBV_ratio	0.786 (0.717 – 0.856)	0.768 (0.54 - 0.99)
